# NEAT: National Epirubicin Adjuvant Trial – toxicity, delivered dose intensity and quality of life

**DOI:** 10.1038/sj.bjc.6604674

**Published:** 2008-09-16

**Authors:** H M Earl, L Hiller, J A Dunn, S Bathers, P Harvey, A Stanley, R J Grieve, R K Agrawal, I N Fernando, A M Brunt, K McAdam, S O'Reilly, D W Rea, D Spooner, C J Poole

**Affiliations:** 1 Oncology Centre, Addenbrookes Hospital, University of Cambridge, Cambridge CB2 0QQ, UK; 2 Warwick Medical School Clinical Trials Unit, University of Warwick, Coventry CV4 7AL, UK; 3 Cancer Research UK Clinical Trials Unit, University of Birmingham, Birmingham B15 2TT, UK; 4 Department of Clinical and Health Psychology, St James's University Hospital, Leeds LS9 7TF, UK; 5 St Chads Pharmacy Department, City Hospital, Birmingham B18 7QH, UK; 6 Arden Cancer Centre, University Hospital, Coventry CV2 2DX, UK; 7 Department of Oncology, Royal Shrewsbury Hospital, Shrewsbury SY3 8XQ, UK; 8 Cancer Centre, Queen Elizabeth Hospital, Birmingham B15 2TH, UK; 9 Staffordshire Oncology Centre, University Hospital of North Staffordshire, North Staffordshire ST4 7LN, UK; 10 Peterborough District Hospital, Cambridgeshire PE3 6DA, UK; 11 Clatterbridge Oncology Centre, Merseyside CH63 4JY, UK

**Keywords:** NEAT, breast cancer, adjuvant chemotherapy toxicity, dose intensity, quality of life

## Abstract

The NEAT trial reported considerable benefit for ECMF (epirubicin followed by cyclophosphamide, methotrexate and 5-fluorouracil) of 28% for relapse-free survival (RFS) and 30% for overall survival (OS), when compared with classical CMF in early breast cancer. To assess tolerability, toxicity, dose intensity and quality of life (QoL) analyses were undertaken. All 2021 eligible patients had common toxicity criteria (CTC), delivered chemotherapy and supportive treatments details and long-term morbidities recorded. The QoL substudy used multiple validated measures. ECMF produced low CTC scores, although higher than CMF for nausea, vomiting, alopecia, constipation, stomatitis (*P*<0.001), infection (*P*=0.001) and fatigue (*P*=0.03). Supportive treatments required, however, were similar across randomised treatments. On-treatment deaths were more common with CMF (13) than ECMF(5). Optimal course-delivered dose intensity (CDDI ⩾85%) was received more often by ECMF patients (83 *vs* 76%: *P*=0.0002), and was associated with better RFS (*P*=0.0006). QoL over 2 years was equivalent across treatments, despite minimally worse side effects for ECMF during treatment. ECMF benefit spanned all levels of toxicity, CDDI and QoL. There are no reported acute myeloid leukaemias or cardiac dysfunctions. ECMF is tolerable, deliverable, and significantly more effective than CMF, with no serious long-term toxicity or QoL detriment.

The National Epirubicin Adjuvant Trial (NEAT) started in 1996, when recorded benefits of adjuvant breast cancer chemotherapy were small, in terms of absolute percentage of advantages in relapse-free survival (RFS) and overall survival (OS) (Peto, 1992, 1998). Attention was therefore also focused on the ‘cost’ of such treatment in terms of toxicity and quality of life (QoL) (Aaronson *et al*, 1993; Sprangers *et al*, 1996). The primary end point of NEAT was to establish whether the anthracycline-based regimen ECMF (epirubicin, cyclophosphamide, methotrexate, 5-fluorouracil) (Bonadonna *et al*, 1995a) would be an improvement on standard classical CMF. With a median follow-up of 4 years, NEAT showed a highly significant 28% advantage in RFS and 30% advantage in OS, with these advantages increasing to 31 and 33%, respectively, when analysed in a meta-analysis with the BR9601 trial (Poole *et al*, 2006). The secondary end points of NEAT were to compare ECMF and CMF in terms of toxicity and QoL. These results are reported here. In addition, we investigated whether ECMF was deliverable with the dose intensity required to achieve the hypothesised improvements in activity over CMF. Hryniuk and Bush (1984) had already analysed the importance of dose intensity in terms of effectiveness.

## Patients and methods

NEAT was a large, randomised, phase III trial comparing optimally scheduled anthracyclines (ECMF) with CMF in women with early-stage breast cancer. The two randomised treatments were: epirubicin (100 mg m^−2^ every 3 weeks) × 4 cycles followed by CMF (cyclophosphamide 100 mg m^−2^ po days 1–14 or cyclophosphamide 600 mg m^−2^ day 1 and 8, i.v.; methotrexate 40 mg m^−2^ days 1 and 8; 5-fluorouracil 600 mg m^−2^ days 1 and 8 every 4 weeks) × 4 cycles (ECMF) *vs* CMF × 6 cycles.

### Toxicity, dose intensity and supportive treatment

For each cycle, data were collected on chemotherapy doses and dates, and common toxicity criteria (CTC) gradings for 10 common toxicities. Hospital admissions and details of supportive treatment were recorded including use of antiemetics, prophylactic antibiotics, growth factors and blood transfusions.

### Deaths during/attributed to chemotherapy and second malignancies

Deaths were recorded on annual follow-up forms. In the absence of disease progression, all deaths during chemotherapy were attributed to chemotherapy although all causes of death were also recorded. Details of second malignancies, including myelodysplasia, experienced during the follow-up period were recorded.

### Quality of life

The QoL substudy was offered to all patients until the accrual target of 500 was met. QoL booklets comprised the EORTC QLQ-C30 (Aaronson *et al*, 1993), the EORTC QLQ-BR23 (Sprangers *et al*, 1996) and a Women's Health Questionnaire (WHQ) (Hunter, 1992). Booklets were completed by participating patients at baseline, mid-chemotherapy, end of chemotherapy and 12 months and 24 months after baseline.

The NEAT trial was approved by a multicentre research ethics committee and by the local research ethics committee at each participating hospital.

## Statistical methods

### Toxicity

#### Worst toxicity grades

For each of the 10 listed toxicities, patient's worst severity during chemotherapy was identified and these compared across treatments using *χ*^2^ tests for trend. Sensitivity analysis was undertaken using data only from patients with full toxicity information on all their received cycles.

#### Incidence of severe toxicity

The number of cycles where each of the 10 listed toxicities was suffered at a severe level (CTC grade 3 or above (two for alopecia)) was compared across treatments using *χ*^2^ tests with continuity corrections. Frequency of supportive treatment use, a substitute measure of toxicity, was summarised. The number of patients deemed to have suffered a severe toxicity at any point throughout their entire treatment course was determined using toxicity reporting and reasons for treatment delay, reduction or hospitalisation. These frequencies were then compared across treatments using *χ*^2^ tests with continuity corrections.

### Chemotherapy

Course delivered dose intensity (CDDI) was calculated as follows: (i) a per drug dose intensity (administered dose per day divided by the planned mg m^−2^ day^−1^); (ii) a per cycle dose intensity (averaging all drug dose intensities planned for that cycle); and (iii) a per patient CDDI was calculated (averaging the above over all planned cycles). Patients with calculable CDDI were compared across treatments using Wilcoxon rank sum tests. The influence of prognostic factors was analysed by multiple regression and logistic regression.

### Quality of life

The C30 questionnaire consists of three scales (15 subscales, 30 questions), the BR23 questionnaire 2 scales (eight subscales, 23 questions) and the WHQ 1 scale (nine subscales, 37 questions). Standardised area under the curve analysis (Qian *et al*, 2000) was undertaken for QoL scales and subscales during the on-treatment period and treatments compared by each scale using O'Brien's global rank procedure (O'Brien, 1984). If significance was found, subscales were investigated with Wilcoxon rank sum tests. To assess long-term QoL, changes from baseline to 1 year and baseline to 2 years were assessed across treatments using O'Brien's global rank procedure and Wilcoxon rank sum tests.

### Prediction of RFS

Kaplan–Meier (Kaplan and Meier, 1958) survival curves were constructed and the log-rank test (Peto *et al*, 1977) was used to assess any differences between CDDI levels. Toxicity, dose intensity and QoL effects on the ECMF benefit over CMF in respect of risk of relapse or death were assessed using forest plots (Early Breast Cancer Trialists' Collaborative Group, 1990).

## Results

Patient characteristics of the 2021 eligible patients, recruited by 111 consultants from 65 UK centres, were balanced across randomised treatments (Supplementary Table 1).

### Toxicity

Toxicity information is available from 12 442 cycles (91% of those with treatment information), from 1952 (97%) patients; 7144 (92%) cycles from 979 ECMF patients and 5298 (91%) cycles from 973 CMF patients. Generally, patients reported low levels of toxicity, although ECMF patients suffered significantly more nausea, vomiting, stomatitis, alopecia, constipation, infection, and fatigue (Tables 1 and 2). The excesses in infections and fatigue, however, were only of a mild nature. The 1543-patient subset with toxicity information on all treatment cycles did not reveal substantially different results.

#### Neutropenic sepsis

Neutropenia was suffered at any grade in 1501 (12%) cycles and infection in 1914 (15%) cycles. Neutropenic sepsis, defined as concurrent neutropenia and infection was reported in 346 (3%) cycles (3% ECMF; 3% CMF, *P*=0.84), by 247 (13%) patients (14% ECMF; 11% CMF, *P*=0.06).

#### Supportive treatment

Supportive treatment information is available for all 13 625 cycles that have been completed by 2012 eligible patients (7777 ECMF, 5848 CMF). Antiemetics were administered in 97% of cycles (98% of cycles by ECMF patients, 97% of cycles by CMF patients) (Supplementary Table 2). The antiemetics used were non-5HT3's (82%), dexamethasone (75%), and 5HT3's (69%). In 12% of cycles, antibiotics were administered (13% during ECMF patients' cycles, 9% during CMF patients' cycles). More antibacterials than antifungals were given (70 *vs* 50% respectively). Only 77 cycles (0.5% of the 13 625) were supplemented by growth factor support, balanced across treatments. Hospitalisation was reported in only 4% of cycles, balanced across treatments, the main reason being sepsis (41%). Blood and platelet transfusions were low in incidence (0.6 and 0.04% of cycles respectively).

### Chemotherapy

#### Number of cycles

Full treatment information was reported for 1985 (98%) patients: 987 (98%) ECMF and 998 (99%) CMF. All planned cycles were received by 891 (90%) ECMF patients and 929 (93%) CMF patients (*P*=0.03). Thirty-two (2%) patients received ⩽2 cycles of chemotherapy; 3 of these receiving no treatment at all. Reasons for not completing the full course were toxicities or unspecified personal reasons.

#### Drug doses received

Oral cyclophosphamide had the lowest overall percentage of dose received (88%) (Table 3), expected when comparing with i.v. drugs, which can be administered with precision to within 1 mg, whereas oral administration is limited because of the fixed tablet size (50 mg). Epirubicin has the highest overall percentage of dose received, perhaps because it is given as the first 4 cycles and therefore less likely to be affected by reductions because of cumulative toxicities.

#### Treatment delays

The median cycle duration was as stated in the protocol: the first 4 cycles on the ECMF arm had median 21 days duration (range 16–84); the last 4 cycles, 28 days (range 20–91); and cycles on the CMF arm had a median 28-day duration (range 17–101). However, CMF patients appear to have suffered marginally more delays despite receiving two fewer cycles (Table 4). Overall, 2376 (18%) cycles were delayed; 1294 (17%) ECMF and 1082 (19%) CMF (*P*=0.008). The median delay was 1 week for both treatments (range 2 days–10 weeks), caused mainly by haematological problems, although longer delays were usually because of patients receiving concurrent radiotherapy. Only 358 (35%) ECMF and 436 (43%) CMF patients suffered no delays.

#### Dose reductions

Overall, 865 (6%) cycles were dose reduced: 400 (5%) cycles by ECMF patients and 465 (8%) by CMF patients (*P*<0.001). The main reason for dose reduction was haematological toxicity. Eight hundred and twelve (80%) ECMF patients and 807 (80%) CMF patients suffered no reductions.

#### Delivered dose intensity

**Figure 1:**
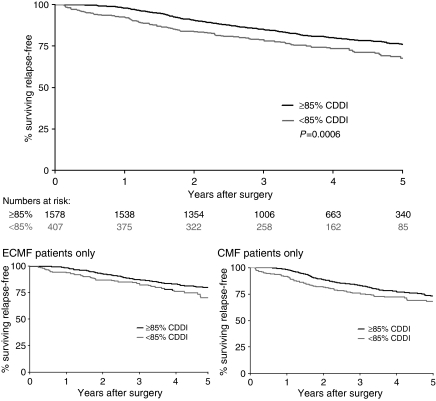
Relapse-free survival by CDDI (<85%, ⩾85%).

Oral cyclophosphamide had the lowest drug dose intensity, with epirubicin and i.v. cyclophosphamide having the highest (Table 3) intensity. There was no apparent decrease of cycle-delivered dose intensity as treatment progressed (Supplementary Figure 1). Median CDDI received was 94% (IQR 87–101%): 94% (range 89–101%) for ECMF and 92% (range 85–100%) for CMF (*P*<0.0001). Eighty percent of patients achieved the ⩾85% CDDI; 83% ECMF patients and 76% CMF patients (*P*=0.0002). Analysis of RFS by patients who received <85% CDDI or ⩾85% CDDI, demonstrates a significant difference in favour of ⩾85% CDDI (*P*=0.0006). This difference is present in both randomised treatment arms (Figure 1).

### Quality of life

The QoL substudy achieved its target sample with 511 patients (25%) (27% ECMF, 23% CMF), which was comparable with the main trial set in terms of patient characteristics, CDDI, RFS and OS. QoL form return was excellent, and balanced across treatments (*P*=0.57). All 511 patients completed baseline forms, 453 (89%) mid-chemotherapy forms, 449 (88%) end-of-treatment forms, 436 (85%) 1-year forms and 411 (80%) 2-year forms. The rate of missing individual question responses was low (4%) and balanced across treatments (4% ECMF, 3.5% CMF). In total, 1595 (71%) questionnaire packs had fewer than five missing responses to the 90 questions. Forty four percent of all missing responses were to sexual questions in the QLQ-BR23 and WHQ. The timing of the 2260 forms is as expected and balanced in accuracy between treatments, except for slightly later end-of-treatment QoL forms for CMF patients (median 19 days late *vs* 13 days late for ECMF patients, *P*=0.04).

**Figure 2:**
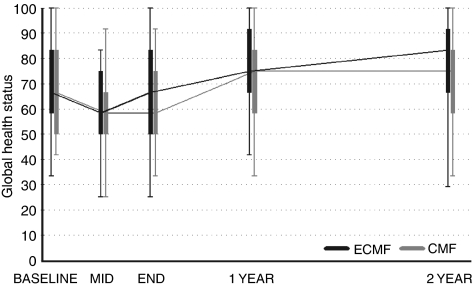
Box and Whisker plots of EORTC QLQ-C30 global QoL domain over time.

Five hundred and one patients had both a completed baseline and mid and/or end-of-treatment questionnaire. The BR23 questionnaire detected a higher frequency of symptoms for ECMF patients during treatment (*P*=0.05). The subscales identified increased ‘*Systemic therapy side effects*’ and ‘*Upset by hair loss*’ (*P*=0.0001 and <0.0001 respectively). No other scales highlighted differences during treatment (C30 Function *P*=0.13, Global Health *P*=0.44 and Symptom *P*=0.50; BR23 Function *P*=0.16, WHQ Global *P*=0.13). The baseline to 1-year change analysis detected differences in both the C30 Global Health and C30 Symptom scale in favour of the ECMF patients (*P*=0.01 and 0.04 respectively). The subscales indicated ECMF patients had greater improvements in global QoL from baseline compared with fewer improvements experienced by CMF patients (*P*=0.01, Figure 2). In addition, CMF patients appeared to be suffering more treatment-related symptoms at 1 year compared with baseline, as opposed to ECMF patients who had similar baseline and 1-year symptom scores. Treatment differences disappeared by 2 years.

### Deaths during/attributed to chemotherapy

Out of the 2021 patients randomised, 18 suffered deaths attributed to chemotherapy (<1%; 5 ECMF: 13 CMF). The most common cause of death was neutropenic sepsis (eight patients) with five fatal pulmonary emboli, and four cerebrovascular accidents. In one patient the cause of death could not be established despite autopsy, and the patient died with a normal blood count. All deaths on treatment in ECMF patients occurred during the CMF phase of the treatment.

### Age and performance status in the prediction of CDDI and toxicity

Age demonstrated no prognostic value for CDDI (*P*=0.62), although performance status (PS) showed a slight trend towards PS 2 patients achieving a lower median CDDI (85% (IQR=65–94) (*P*=0.07)). Age had no effect on CDDI categorised as <85 or ⩾85% (*P*=0.65), but PS had a significant prognostic value. PS 0 had 80% patients with CDDI ⩾85%, PS 1 had 77% patients and PS 2 had 50% (*P*=0.05). Predicting severe toxicity suffered during treatment demonstrated that age >50 years and PS 2 predicted for a higher incidence of severe neutropenia (*P*=0.003 and *P*<0.0001 respectively).

### CDDI, toxicity and QoL in the prediction of RFS

Analysis of the interaction between toxicity, CDDI, and QoL with the RFS benefits observed for all ECMF patients, show no statistically significant heterogeneity or trends. The benefit of ECMF over CMF holds true for all patients in the study, over the full range of experiences of toxicities, CDDI and QoL.

### Second malignancies and cardiac morbidity

Forty-seven patients have reported second malignancies (2%). Over half of these are second primaries in the contralateral breast (Supplementary Table 3). Only one patient has been reported with acute leukaemia, which was pro-myelocytic in type, and judged unrelated to chemotherapy. There are no reports of cardiac morbidity.

## Discussion

The results of the NEAT study have been published in detail elsewhere (Poole *et al*, 2006) but in summary, show considerable advantages in terms of RFS (28%) and OS (30%) for patients receiving ECMF compared with CMF. ECMF demonstrated low rates of toxicity, although more than were recorded in CMF patients. Interestingly, more deaths during treatment occurred on the CMF arm and deaths that occurred on the ECMF arm were all during CMF, although we were unable to identify any early warning indicators for treatment-related deaths. A continued high level of vigilance is required in all patients receiving adjuvant chemotherapy for breast cancer.

Bonadonna *et al* (1995b) reported total dose delivered in the retrospective analysis of their original CMF *vs* control adjuvant breast cancer trial and demonstrated that when total doses were below 85% there was no advantage compared with the ‘no treatment’ control arm. Hryniuk's work on delivered dose intensity added increased sophistication to this important emerging concept (Hryniuk and Bush, 1984). The median CDDI was high in NEAT at 94%, and optimal CDDI (⩾85%) was more often achieved in ECMF than CMF patients (83 *vs* 76% respectively; *P*=0.0002). In addition, significantly higher RFS was shown for optimal (⩾85%) CDDI compared with reduced CDDI (*P*=0.0006), and this held for both the ECMF and CMF arms. These data confirm the deliverability of ECMF.

It is often said that older patients and those with low performance status suffer more toxicity from chemotherapy, and therefore dose intensity may be compromised. Although this is undoubtedly true for the 65 years plus age group, in this study we were able to analyse whether being under or over the age of 50 years made any difference to toxicity, CDDI, or QoL. Multiple regression analysis was carried out to look for prediction by age and performance status (PS) of CDDI. It was only PS 2 patients who showed a slight trend towards achieving a lower median CDDI (85% (IQR=65–94) (*P*=0.07)). Logistic regression confirmed this showing that only 50% of PS 2 patients achieved an optimal CDDI ⩾85% (*P*=0.05). Age >50 years and PS 2 predicted for a higher incidence of neutropenia (*P*=0.003 and *P*<0.0001 respectively) only, out of all possible toxicities.

Analysis of interaction between toxicity, CDDI, and QoL with treatment effect (ECMF *vs* CMF) with tests for heterogeneity and trends, show no statistically significant effects. This is important as it shows that the benefit of ECMF over CMF holds true for all patients in the study, over the full range of experiences of different toxicities, CDDI and QoL.

Quality-of-life assessment is an important part of the analysis of results in cancer clinical trials. The collection of over 80% of longitudinal data in more than 500 patients makes it possible for us to draw some important conclusions. Although there was some minor and temporary relative worsening of quality of life for patients in the ECMF arm, the improvements in RFS and OS far outweighed this small and transitory reduction in QoL.

In conclusion, the tolerability and acceptability of the NEAT treatment regimens were critical end points. Despite differences in acute toxicities and short-term QoL between ECMF and CMF, both regimens were shown to be tolerable, with the majority of patients receiving ⩾85% CDDI. Only low levels of supportive treatments and hospitalisations were necessary, and both regimens were associated with similar long-term QoL outcomes. The benefit for ECMF over CMF holds true for all groups of patients regardless of toxicity, CDDI or QoL.

## Figures and Tables

**Table 1:** Worst severity suffered by patient, throughout all cycles for each stated toxicity

		**ECMF (*n*=979)**		**CMF (*n*=973)**		
**Toxicity**	**Grade**	***N***	**%**	***N***	**%**	P*
Nausea^a^	0	134	13	244	25	<0.001
	1/2	697	71	657	67	
	3	146	15	69	7	
Vomiting	0	451	46	609	63	<0.001
	1/2	412	42	327	33	
	3/4	113	11	36	3	
Stomatitis	0	283	29	437	45	<0.001
	1/2	634	64	507	52	
	3/4	61	6	27	3	
Alopecia^a^	0	87	9	234	24	<0.001
	1	72	7	472	48	
	2	820	84	263	27	
Constipation	0	516	53	674	69	<0.001
	1/2	390	40	271	28	
	3/4	62	6	24	2	
Infection	0	418	43	505	52	0.001
	1/2	465	47	397	41	
	3/4	64	7	51	5	
Fatigue^a^	0	131	13	154	16	0.03
	1/2	638	65	640	65	
	3	204	21	177	18	
Neutropenia	0	652	67	676	69	>0.99
	1/2	162	16	140	14	
	3/4	151	15	143	15	
Thrombocytopenia	0	916	93	907	93	>0.99
	1/2	54	6	55	6	
	3/4	8	1	10	1	
Diarrhoea	0	541	55	512	53	>0.99
	1/2	380	39	401	41	
	3/4	56	6	58	6	

^*^P-values after Bonferroni correction from tests on full breakdown of toxicity grades.

a CTC gradings for nausea and fatigue have a maximum of three and, for Alopecia, a maximum of two.

**Table 2:** Incidences of severe^a^ toxicity suffered by cycle and by patient

	**ECMF**		**CMF**		**Total**	
	***N***	**%**	***N***	**%**	***N***	**%**
*Number of cycles*	(*n*=7144)		(*n*=5298)		(*n*=12442)	
Nausea	204	2.9	89	1.7	293	2
Vomiting	172	2.4	45	0.9	217	2
Stomatitis	86	1.2	34	0.6	120	1
Alopecia	3850	53.9	769	14.5	4619	37
Constipation	100	1.4	37	0.7	137	1
Infection	76	1.1	62	1.2	138	1
Fatigue	429	6.0	326	6.2	755	6
Neutropenia	275	3.9	227	4.3	502	4
Thrombocytopenia	10	0.1	11	0.2	21	1
Diarrhoea	78	1.1	87	1.6	165	1
Neutropenic Sepsis^b^	201	3	145	3	346	3
						
*Number of patients* c	(*n*=1004)		(*n*=1008)		(*n*=2012)	
Nausea	169^***^	17	85	8	254	13
Vomiting	129^***^	13	48	5	177	9
Stomatitis	70^**^	7	33	3	103	5
Alopecia	821^***^	82	263	26	1084	54
Constipation	63^***^	6	26	3	89	4
Infection	208	21	163	16	371	18
Fatigue	212	21	183	18	395	20
Neutropenia	334	33	347	34	681	34
Thrombocytopenia	26	3	21	2	47	2
Diarrhoea	72	7	77	8	149	7

Asterisks indicate significantly higher number of patients, after Bonferroni correction (^*^P<0.05, ^**^P<0.01, ^***^P<0.001).

a Severe=reported CTC grade ⩾3 (⩾2 for alopecia).

b Neutropenic Sepsis episodes inferred from incidences, of any grade, of neutropenia and infection reported in the same cycle.

c Gleaned from a reported severe grade or cause of treatment delay, reduction or hospitalisation.

**Table 3:** Drug doses received and drug delivered dose intensity (dDDI)

	**ECMF**		**CMF**	
**Drugs**	**% dose received**	**Median (IQR) dose intensity**	**% dose received**	**Median (IQR) dose intensity**
Epirubicin	99	100 (95–105)	100^a^	99 (99–100)
Oral Cyclophosphamide	89	88 (78–105)	88	87 (77–104)
i.v. Cyclophosphamide	96	100 (93–104)	96	100 (92–104)
Methotrexate	97	99 (92–104)	97	99 (91–104)
5-FU	97	99 (93–104)	97	99 (91–104)

a Based on two CMF patients who received Epirubicin through protocol violations.

**Table 4:** Treatment delays and dose reductions per treatment cycle^a^ on 13 454 cycles

	**ECMF (*n*=7658)**		**CMF (*n*=5796)**		**Total (*n*=13 454)**	
	***N***	**%**	***N***	**%**	***N***	**%**
Delays suffered within cycle	1294	17	1082	19	2376	18
*Length of delay (days)*						
Median (IQR)	7 (5–7)		7 (7–7)		7 (6–7)	
Range	2–63		2–73		2–73	
						
*Reasons for delay*						
Haematological	400	31	446	41	846	36
Admin/personal reasons	130	10	98	9	228	10
Radiotherapy	85	7	69	6	154	6
Other	156	12	112	10	268	11
Unknown	529	41	371	34	900	38
						
Dose reduction within cycle	400	5	465	8	865	6
						
*Reasons for dose reduction*						
Haematological	119	30	124	27	243	28
Admin/personal reasons	12	3	20	4	32	4
Other	94	23	95	20	189	22
Unknown	175	44	228	49	403	46

a Delay defined as >1 day late from previous cycle; Reduction defined as <85% of expected doses. Some cycles were delayed or reduced for more than one reason.
