# Supplementary figures and images for: NEAT: National Epirubicin Adjuvant Trial – toxicity, delivered dose intensity and quality of life
Source: Br J Cancer. 2008 Sep 16;99(8):1226–31. doi: 10.1038/sj.bjc.6604674 (PMC2570521; doi:10.1038/sj.bjc.6604674)

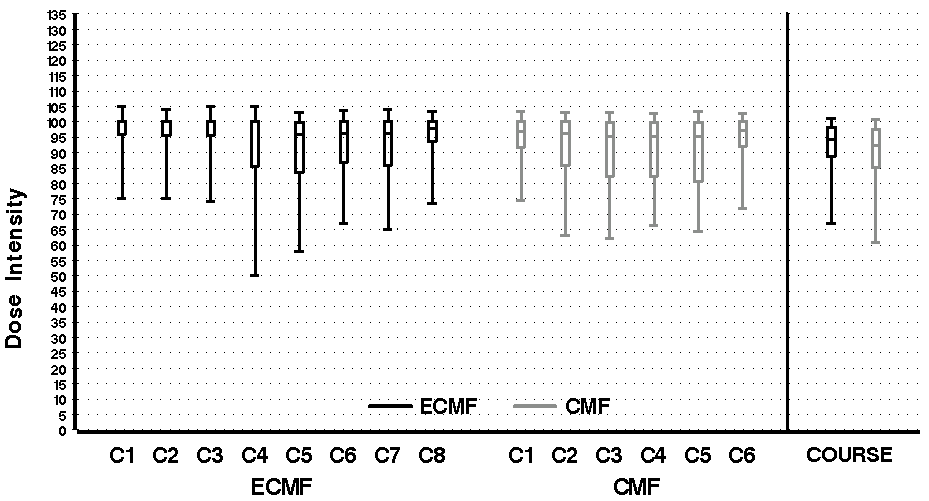

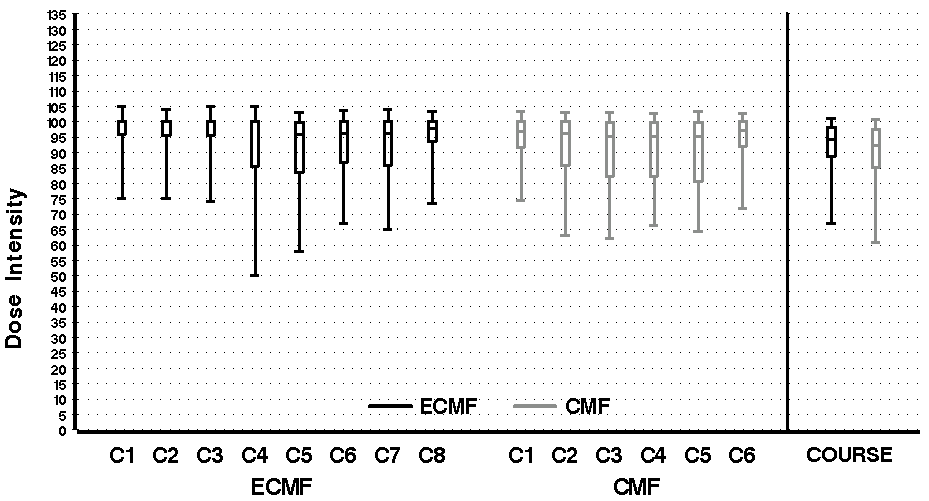

Supplement: Supplementary Figure 1 [file 6604674x1.doc]
