# Supplementary material for: NEAT: National Epirubicin Adjuvant Trial – toxicity, delivered dose intensity and quality of life
Source: Br J Cancer. 2008 Sep 16;99(8):1226–31. doi: 10.1038/sj.bjc.6604674 (PMC2570521; doi:10.1038/sj.bjc.6604674)
Supplement: Supplementary Tables and Figure Legend [file 6604674x2.doc]

**Supplementary Table 1.** Patient Characteristics - 2021 eligible patients

|  | **ECMF (N=1009)** | | **CMF (N=1012)** | | **Overall(N=2021)** | |
| --- | --- | --- | --- | --- | --- | --- |
|  | **N** | **%** | **N** | **%** | **N** | **%** |
| **Age** <50 years old | 622 | 62 | 606 | 60 | 1228 | 61 |
| >50 years old | 387 | 38 | 406 | 40 | 793 | 39 |
| **Menopausal status**  Pre | 488 | 48 | 486 | 48 | 974 | 48 |
| Peri | 86 | 9 | 97 | 10 | 183 | 9 |
| Post | 365 | 36 | 373 | 37 | 738 | 37 |
| Unknown | 70 | 7 | 56 | 5 | 126 | 6 |
| **Performance Status** |  |  |  |  |  |  |
| 0 – Fully active | 706 | 70 | 700 | 69 | 1406 | 69 |
| 1– Restricted | 192 | 19 | 206 | 20 | 398 | 20 |
| 2 – Capable self care | 4 | 1 | 6 | 1 | 10 | 1 |
| Unknown | 107 | 10 | 100 | 10 | 207 | 10 |
| Type of Surgery Mastectomy | 505 | 50 | 513 | 50.5 | 1018 | 50.5 |
| Breast Conserving Surgery | 500 | 49.5 | 496 | 49 | 996 | 49 |
| Unknown | 4 | 0.5 | 3 | 0.5 | 7 | 0.5 |
| **Surgery to randomisation** N | 1002 | | 1007 | | 2009 | |
| (days) Median (IQR) | 25 (20 – 33) | | 24 (19 – 33) | | 25 (20 – 33) | |

**Supplementary Table 2.** Reported Supportive Treatment Use and Hospital Admissions on 13,625 Cycles

|  | **ECMF**  **(n=7,777)** | | **CMF (n=5,848)** | | **Total** | |
| --- | --- | --- | --- | --- | --- | --- |
|  | **N** | **%** | **N** | **%** | **N** | **%** |
| Reported **anti-emetic** use | 7615 | 98 | 5650 | 97 | 13265 | 97 |
|  |  |  |  |  |  |  |
| Type of anti-emetic 5HT3 | 6022 | 79 | 3165 | 56 | 9187 | 69 |
| non5HT3 | 6158 | 81 | 4776 | 85 | 10934 | 82 |
| Dexamethasone | 6013 | 79 | 3935 | 70 | 9948 | 75 |
|  |  |  |  |  |  |  |
| Reported **prophylactic antibiotics** use | 1040 | 13 | 554 | 9 | 1594 | 12 |
|  |  |  |  |  |  |  |
| Type of antibiotic Anti-Bacterial | 759 | 73 | 356 | 64 | 1115 | 70 |
| Anti-Fungals | 543 | 52 | 251 | 45 | 794 | 50 |
|  |  |  |  |  |  |  |
| Reported **growth factor** use | 38 | 0.5 | 39 | 0.5 | 77 | 0.5 |
|  |  |  |  |  |  |  |
| Type of growth factor GCSF | 38 | 100 | 39 | 100 | 77 | 100 |
| GMCSF | 1 | 3 | 2 | 5 | 3 | 4 |
|  |  |  |  |  |  |  |
| Reported **hospital admissions** | 325 | 4 | 255 | 4 | 580 | 4 |
|  |  |  |  |  |  |  |
| Reason for hospitalisation^ Sepsis | 128 | 39 | 109 | 43 | 237 | 41 |
| Blood Transfusion | 27 | 8 | 28 | 11 | 55 | 9 |
| Nausea/Vomiting | 36 | 11 | 17 | 7 | 53 | 9 |
| Infection | 23 | 7 | 15 | 6 | 38 | 7 |
| Neutropenia | 12 | 4 | 17 | 7 | 29 | 5 |
| Diarrhoea | 16 | 5 | 15 | 6 | 31 | 5 |
| Other | 91 | 28 | 74 | 29 | 165 | 28 |
| Unknown | 13 | 4 | 13 | 5 | 26 | 4 |

^ Some cycles had multiple reasons for hospitalisation

**Supplementary Table 3.** Sites of the 47 Second Primaries

|  | **ECMF (n=25)** | **CMF (n=22)** | **Total (n=47)** |
| --- | --- | --- | --- |
| **Contralateral Breast** | 15 | 9 | 24 |
| Endometrial | 1 | 3 | 4 |
| **Ovarian** | 0 | 3 | 3 |
| **Non-Small Cell Lung** | 2 | 1 | 3 |
| **Head and Neck** | 1 | 2 | 3 |
| **Basal Cell** | 2 |  | 2 |
| **Lymphoma** |  | 1 | 1 |
| **Colon** |  | 1 | 1 |
| **Bone** |  | 1 | 1 |
| **Pouch of Douglas** |  | 1 | 1 |
| **APML** | 1 |  | 1 |
| **Renal cell** | 1 |  | 1 |
| **Melanoma** | 1 |  | 1 |
| **Rectum** | 1 |  | 1 |

**Supplementary Figure 1**. Box and Whisker plots of Cycle Delivered Dose Intensity (cDDI) and Course Delivered Dose Intensity (CDDI)
